# Exploring Ethnic Inequalities in Admission to Russell Group Universities

**DOI:** 10.1177/0038038515575859

**Published:** 2015-05-12

**Authors:** Vikki Boliver

**Affiliations:** Durham University, UK

**Keywords:** ethnicity, fair access, Russell Group universities

## Abstract

This article analyses national university applications and admissions data to explore why ethnic minority applicants to Russell Group universities are less likely to receive offers of admission than comparably qualified white applicants. Contrary to received opinion, the greater tendency of ethnic minorities to choose highly numerically competitive degree subjects only partially accounts for their lower offer rates from Russell Group universities relative to white applicants with the same grades and ‘facilitating subjects’ at A-level. Moreover, ethnic inequalities in the chances of receiving an admissions offer from a Russell Group university are found to be greater in relation to courses where ethnic minorities make up a larger percentage of applicants. This latter finding raises the possibility that some admissions selectors at some Russell Group universities may be unfairly rejecting a proportion of their ethnic minority applicants in an attempt to achieve a more ethnically representative student body.

## Introduction

Despite being more likely than their white British counterparts to enrol in higher education generally (Modood, 2012), British students from black Caribbean, black African, Pakistani and Bangladeshi ethnic backgrounds continue to be strikingly under-represented in the UK’s most prestigious universities (Business in the Community, 2010). Indeed, as Alan Milburn, Chair of the Independent Commission on Social Mobility, pointed out in his report entitled *University Challenge*:

… there are more young men from black backgrounds in prison in the UK than there are UK-domiciled undergraduate black male students attending Russell Group institutions. (Milburn, 2012: 21)^1^

In the same vein, the Prime Minister, David Cameron, has criticized Oxford University for its ‘disgraceful’ record of admitting British ethnic minority students (Porter et al., 2011). Cameron was referring to evidence obtained through Freedom of Information requests by David Lammy MP, who, in an article for *The Guardian* entitled ‘The Oxbridge whitewash’ wrote:

Just one British black Caribbean student was admitted to Oxford last year [in 2009]. That is not a misprint: one student. Merton College, Oxford, has not admitted a single black student for five years. At Robinson College, Cambridge, a white applicant is four times more likely to be successful than a black applicant. […] Applications are being made but places are not being awarded. (Lammy, 2010)

David Lammy’s original claim was quickly rebutted by Oxford University Pro-Vice-Chancellor Dr Sally Mapstone, who, in an article that appeared in *The Guardian* a few days later, countered that:

School attainment is the single biggest barrier to getting more black students to Oxford. […] If Mr Lammy thinks Oxford and Cambridge’s data makes ‘shocking reading’, he should try the national figures. In 2009, 29,000 white students got the requisite grades for Oxford (AAA excluding general studies), compared to just 452 black students. (Mapstone, 2010)

As Dr Mapstone points out, prospective university students from black and certain other ethnic minority backgrounds tend to have poorer A-level grades than white applicants on average (BIS, 2013; Connor et al., 2004), which in turn largely accounts for their lower rates of application to highly selective universities (Boliver, 2013). However, when university applicants from black and other ethnic minority backgrounds do apply to Oxford University or to Russell Group universities more generally, they are substantially less likely to be offered places than white applicants with comparable A-level qualifications (Boliver, 2004, 2013; Noden et al., 2014; Shiner and Modood, 2002; Taylor, 1992; Zimdars et al., 2009).^2^

Exactly why ethnic minority applicants are so disadvantaged in the competition for places at more prestigious UK universities, relative to their comparably qualified white peers, has yet to be established. One possibility, of course, is that of ethnic bias in the university admissions process. Perhaps because universities tend to be seen by those who work in them as particularly liberal and progressive places, prejudice and discrimination are often assumed to be absent in higher education (Back, 2004; Pilkington, 2012; Turney et al., 2002). However, research on the experiences of ethnic minority students and staff in UK higher education institutions finds racism to be commonplace (Equality Challenge Unit, 2011; National Union of Students, 2011). Recent photo campaigns highlight the concerns of some ethnic minority students at Harvard University and Oxford University that their ‘presence is questioned’ (http://itooamharvard.tumblr.com/) and that they ‘are made to feel different’ (http://itooamoxford.tumblr.com/) by others within their academic communities. UK university students from ethnic minority backgrounds have been found to be more dissatisfied with the assessment process than their white counterparts, especially when work is not marked anonymously (National Union of Students, 2011; Surridge, 2008). Moreover, ethnic minority students have been shown to receive poorer marks at degree level than white students with the same levels of prior attainment (Broecke and Nicholls, 2007; HEFCE, 2014).

It seems likely that ethnic minorities’ poorer experiences of higher education are partly due to racial prejudice and stereotyping. Few studies have directly explored the biases of university academics in the UK, but numerous school-based studies show that some teachers have substantially lower expectations for ethnic minority students than those students’ actual ability and attainment would warrant (Strand, 2012; van den Berg et al., 2010), even for ethnic minority students from middle-class families (Gillborn et al., 2012). The evidence from research in UK schools also suggests that while high-achieving white middle-class students tend to be viewed as ‘ideal pupils’, high-achieving Chinese students are ‘pathologized’ as too quiet and too passive, and low-achieving black and Muslim students are ‘demonized’ as loud, challenging and hypersexualized (Archer, 2008; see also Rollock, 2007).

It would be surprising if similar prejudices and stereotypes were not operating in higher education institutions too. Indeed, the Equality Challenge Unit, established in 2006 to help advance equality and diversity in higher education institutions, recently highlighted the need for university staff to guard against the influence of unconscious biases relating to various social groups on the judgements and decisions they make in the course of their daily work including in relation to university admissions (Equality Challenge Unit, 2013). There is no direct evidence on this issue in relation to UK university admissions at present, but experimental evidence from the US suggests that college professors are less likely to respond to unsolicited emails from prospective graduate students if the sender’s name indicates that they are from an ethnic minority (Milkman et al., 2012).

UK universities have clearly come under growing political and legal pressure since the mid-2000s to ensure the fair and consistent treatment of applicants from different ethnic and other social groups throughout the university admissions process (Schwartz, 2004; Universities UK, 2003). These principles are enshrined in UK equalities legislation, most notably the Equality Act passed in 2010, and the Public Sector Equality Duty created by the Act, which requires universities as public institutions to ensure not only that they do not unlawfully discriminate against applicants on the basis of any of nine ‘protected characteristics’, including ‘race’/ethnicity, but also that their policies and practices actively advance equality of opportunity and foster good relations between people from different groups (Equality Challenge Unit, 2012; Equality and Human Rights Commission, 2012). As a key means of ensuring equal treatment, universities must apply admissions selection criteria consistently (Quality Assurance Agency, 2006), and they are expressly prohibited from offering more or less favourable terms of admission to applicants on the basis of certain protected characteristics, including ethnic background (Equality and Human Rights Commission, 2013).

Of course it may be that ethnic differences in offer rates have a far more benign cause. One major contender suggested by Dr Mapstone is the greater tendency of ethnic minority applicants to apply to heavily oversubscribed courses:

Our own recent analysis shows that subject choice is a major reason for the lower success rate. Black students apply disproportionately for the three most oversubscribed subjects: 44% of black applicants, compared to just 17% of white applicants. (Mapstone, 2010)

The same explanation for ethnic group differences in offer rates appeared on the websites of Oxford and Cambridge Universities in December 2010 (Cambridge University, 2010; Oxford University, 2010), and again on the Russell Group website in March 2013 (Russell Group, 2013) and the Oxford University website in 2014 (Oxford University, 2014). Moreover, this contention has been repeated by the Director of the Office for Fair Access, Professor Les Ebdon, first in the *Times Education Supplement* in November 2012, and subsequently in *The Sunday Times*:

One of the interesting things I have discovered is that one of the underlying reasons for the under-representation of ethnic minorities in some highly selective universities is because they apply predominantly for medicine and law, both highly competitive courses … (Les Ebdon, quoted in *The Sunday Times*; Griffiths, 2013)

As these comments illustrate, it is becoming the received wisdom that ethnic minority students are less likely to be admitted to the UK’s most prestigious universities than their comparably qualified white peers in large part because they are more likely to apply to courses that are in high demand. However, at the time of writing, neither Oxford University nor Cambridge University have published their research on this issue in any detail, and what little published evidence there is does not support this claim.

What the evidence available to date does make clear is that, at undergraduate level, ethnic minority students are statistically over-represented among those studying certain subjects, most notably Medicine and Dentistry, Law, Computer Science, Business Studies and Mathematics (Connor et al., 2004: 47–8). Correspondingly, ethnic minorities are statistically under-represented among undergraduates in fields such as the Humanities, Education, Languages, the Creative Arts and the Physical Sciences (Connor et al., 2004: 47–8). This pattern replicates that seen at A-level (Vidal Rodeiro, 2009) and is consistent with the idea that ethnic minority students and their families strongly prefer subjects that lead directly to traditional professional occupations or that develop the skills needed to be successfully self-employed. Indeed, compared to their white peers, ethnic minorities are less likely to cite personal interest as the reason for their subject choice and are more likely to cite employment and career plans (Connor et al., 2004: 51). These subject preferences appear to be guided at least in part by a perception that certain occupations offer a greater degree of protection from anticipated racial discrimination in the labour market (Gill, 2009).

While there is abundant evidence that ethnic minority students are relatively concentrated in certain subject areas, including those that are especially competitive with regard to university admissions, no study to date has provided clear and direct evidence that it is this that accounts for the ethnic differences in offer rates at more prestigious UK universities that remain after controlling for differences in applicants’ prior attainment. On the contrary, previous studies have found that ethnic minority applicants to prestigious Russell Group universities are less likely to receive offers than their equivalently qualified white peers even after controlling for applicants’ chosen degree subject areas (Boliver, 2004, 2013; Noden et al., 2014). Existing research also shows that even very highly-qualified applicants from ethnic minority backgrounds are substantially less likely than their white counterparts to be offered places on some of the most numerically competitive courses at Oxford and Cambridge universities, notably Medicine (Ball and Parel, 2013; Boliver, 2014; Parel and Ball, 2013). However, these previous studies do not control directly for variation in the numerical competitiveness of entry to different subject areas at different universities. The present study, in contrast, sets out to do just that.

The present study also sets out to test a further hypothesis: namely that, controlling for the numerical competitiveness of different courses and for applicants’ prior attainment, ethnic differences in the chances of receiving an offer from a Russell Group university widen as the percentage of ethnic minority applicants to particular degree subject areas at particular institutions increases. Because ethnic minorities apply disproportionately to certain degree subjects, because they are relatively residentially concentrated (Craig, 2012), and because they are more likely than their white counterparts to apply to universities close to home (Gibbons and Vignoles, 2009; Khambhaita and Bhopal, 2013), ethnic minority applicants tend to make up rather higher proportions of applicants to certain degree subject areas at certain universities compared to their representation in the UK population at large (Connor et al., 2004). Where this is the case, it is possible that admissions selectors may be rejecting ethnic minority applicants at higher rates than comparably qualified white applicants in order to bring the proportion of ethnic minority entrants down to a figure closer to, say, their actual or perceived proportion of the wider population. Correspondingly, where ethnic minority applicants are relatively few in number, admissions selectors may be offering places to ethnic minority applicants at rates that are the same as, or perhaps even higher than, the rates at which they offer places to comparably qualified white applicants.

One possible motivation for such a practice could be the perception that admitting ‘too many’ students from ethnic minority and other non-traditional backgrounds erodes institutional prestige – something that non-traditional university students themselves recognize (Reay et al., 2001). Alternatively it could be that admissions selectors at Russell Group universities are making decisions based on a conception of fairness as ultimate representativeness rather than fairness as equal treatment. Suggestive of this, a case study of the University of Leeds found that a number of academic departments gauged how well they were doing in terms of equal opportunities by comparing the representation of ethnic minorities among their students and staff to a figure of 5.5 per cent derived from the 1991 census (Turney et al., 2002: 37). Equating fairness with ultimate representativeness in this way may seem an attractive option given that a diverse student body is likely to expose students to a wider range of experiences and viewpoints which may enhance not only their learning but also their civic-mindedness (Anderson, 2010; Gurin et al., 2002). However, it is important to be clear that diversity and representativeness are not synonymous, and that the benefits of diversity can still be had even if the student body is not perfectly representative of the wider population. Indeed, we might even prefer a diverse study body that is imperfectly rather than perfectly representative if this makes it possible to have equal treatment of applicants at the point of admissions as well. Simply equating fairness with ultimate representativeness is clearly problematic given that a group whose members apply in high numbers could end up being well-represented among students relative to their proportion of the wider population *despite* being treated unfairly at the point of admission. Equating fairness with ultimate representativeness is also problematic because it is not obvious what we should seek to make representative (is it each degree course, each university or the population of university students nationally?), nor is it obvious what we should seek to make it representative of (the national population, or a more local one?). In any case, disproportionately rejecting ethnic minority applicants because they make up a disproportionately large share of applicants would not only be unfair but also potentially unlawful under the provisions of the 2010 Equality Act.

Previous research on admission to Oxford University found that the courses to which ethnic minorities applied in disproportionately large numbers were those in which they experienced the largest admissions disadvantage relative to white applicants (Boliver, 2004). The present study sets out to explore whether ethnic inequalities in admissions chances are linked to the percentage of ethnic minority applicants to courses at prestigious Russell Group universities more generally.

## Data and Methods

Ethnic inequalities in admission to Russell Group universities are explored in this article using individual level applicant data supplied by the Universities and Colleges Admissions Service (UCAS). UCAS is the administrative body that assists universities in handling applications to almost all full-time higher education courses in the UK. Applicants using the UCAS system can apply for up to five courses simultaneously. Applications are passed on to the universities concerned where admissions selectors, who are often academics based in the relevant department but may be administrators in centralized admissions offices, decide whether or not to make the applicant an offer of a place. Admissions selectors base their decisions on a range of criteria, including predicted or actual grades at A-level or in equivalent qualifications; experience of studying subjects that are formal prerequisites of the course; achieved grades at GCSE; applicants’ personal statements; teacher references; and in some cases university-administered tests and interviews. Most offers are made on a conditional basis, requiring the applicant to subsequently achieve the academic entry requirements of their chosen course in national examinations (e.g. A-levels) taken later in the academic year.

The UCAS dataset analysed in this article comprises a 10 per cent random sample of all ‘home’ applicants to full-time undergraduate degree courses at UK universities commencing in 2010/2011, 2011/2012 and 2012/2013. The working sample contains information on 68,632 UCAS candidates who collectively submitted 151,281 applications to Russell Group universities. Applications, rather than applicants, are taken as the unit of analysis.

A series of binary logistic regression models are used to estimate the comparative odds of an application to a Russell Group university being met with an offer of admission, rather than being rejected, if the candidate is from an ethnic minority background rather than from the white group. Information about ethnicity is based on self-reports by applicants on their UCAS form. It is important to note that information about the ethnic origin of applicants is not communicated to admissions selectors at any point during the admissions decision-making process. However, it seems likely that admissions tutors would have an idea of the ethnic origin of some applicants from seeing applicants’ names printed on their UCAS forms, and perhaps also from knowing applicants’ home addresses, the schools they attended, and the substance of their personal statements and references.

Applicants’ prior attainment is measured using information about their actual attainment at A-level or in equivalent qualifications as communicated to UCAS by the exam boards. The analysis reported below includes applicants’ grades at A-level (excluding General Studies) or their UCAS tariff point equivalent if they hold qualifications other than A-level; and whether or not they had studied at A-level each of eight subjects identified by the Russell Group as ‘facilitating’ access to Russell Group universities, namely Biology, Chemistry, English Literature, Geography, History, Languages, Mathematics and Physics (Russell Group, 2012). It is important to note that this data refers to applicants’ actual attainment at A-level or equivalent whereas admissions selectors usually base their decisions on information about applicants’ predicted A-level attainment and their actual attainment at GCSE. Unfortunately, due to restrictions placed by UCAS on the supply of data to external researchers, it has not been possible to include predicted A-level attainment or actual GCSE attainment in the analysis that follows. It should also be noted that it has only been possible to control individually for eight generally ‘facilitating’ A-level subjects, rather than for specific combinations of A-level subjects which are prerequisites for admission to particular degree programmes. Again, this is due to data supply restrictions, which mean that instead of information on the specific degree courses to which applicants are seeking entry, the dataset only contains information about the broad degree subject areas to which applicants applied. Based on the Joint Academic Coding System (JACS) method of classifying degree subjects, a total of 23 degree subject areas (e.g. ‘Medicine & Dentistry’, ‘Biological Sciences’, etc.) are distinguishable in the dataset.

The numerical competitiveness of applicants’ chosen degree programmes is measured by calculating the initial rejection rate for each degree subject area at each Russell Group university present (in anonymized form) in the dataset. The 23 degree subject areas multiplied by the 20 universities that were members of the Russell Group during the period under consideration gives a total of 460 possible (and 398 actual) combinations for which the numerical competitiveness variable could be calculated. As noted above, the 23 degree subject areas identifiable in the data are relatively broad categories, and each comprises a large number of specific degree programmes with varying levels of numerical competitiveness. As a result, the numerical competitiveness variable used in the analysis that follows is subject to a certain degree of unmeasured heterogeneity, and so the extent to which numerical competitiveness accounts for ethnic group differences in the chances of receiving an offer of a place at a Russell Group university may be under- or over-estimated. However, after implementing a control for numerical competitiveness in this relatively crude way, if ethnic group differences in the chances of an offer from a Russell Group university remain substantial, we can be reasonably confident that the lower offers rates for ethnic minorities are not wholly due to disproportionate rates of application to oversubscribed courses.

Finally, the percentage of ethnic minority applicants is calculated from the data at hand and is simply the percentage of applicants to each degree subject area at each university who self-identified as an ethnic minority rather than as white. This variable is centred on the sample mean of 21.4 per cent. As with the variable for numerical competitiveness, this variable is likely to contain some degree of unmeasured heterogeneity and so its importance as a factor that mediates ethnic group difference in admissions chances may also be under- or over-estimated.

## Results

Before proceeding to the results of the multivariate analysis, Table 1 presents some descriptive statistics regarding the features of applications submitted to Russell Group universities by UCAS candidates from different ethnic groups. The first column of figures begins by showing that 54.7 per cent of applications submitted by white candidates are met with offers of admission, whereas offer rates are much lower for applicants from black Caribbean (29.6%), black African (21.9%), Pakistani (30.3%) and Bangladeshi (31.2%) backgrounds, and even for applicants from Indian (43.1%), Chinese (49.6%), Mixed (47.8%) and Other (34.9%) ethnic backgrounds.

**Table 1.** Descriptive statistics for applications submitted to Russell Group universities for entry in 2010/2011, 2011/2012 and 2012/2013.

Ethnicity of applicant	Mean offer rate	Mean A-level points achieved by applicant	Mean numerical competitiveness of chosen degree subject area at chosen university	Mean percentage of ethnic minority applicants to chosen degree subject area at chosen university	Sample size
White	54.7	348	47.2	18.6	117,732
Black Caribbean	29.6	303	56.2	32.0	1,250
Black African	21.9	310	58.6	33.2	5,782
Pakistani	30.3	318	57.4	32.4	4,265
Bangladeshi	31.2	311	59.4	35.9	1,177
Indian	43.1	360	57.8	33.4	6,673
Chinese	49.6	413	54.2	29.4	2,147
Mixed	47.8	356	51.7	24.2	5,599
Other	34.9	346	58.1	33.9	5,101

Column 2 of Table 1 describes ethnic group differences in applicants’ prior academic attainment. Here, it can be seen that A-level qualified applicants to Russell Group universities from black Caribbean, black African, Pakistani and Bangladeshi backgrounds have A-level point scores that are 30–45 points lower on average than their white peers, the equivalent of BBB at A-level for black Caribbean applicants compared to AAB for white applicants. Applicants from the Indian, Mixed and Other ethnic groups, in contrast, have similar average A-level point scores to white applicants, whereas Chinese applicants have A-level point scores that are 60 points higher on average, equivalent to A*A*A at A-level.

Column 3 of Table 1 describes the average numerical competitiveness of the degree programmes at the Russell Group universities to which candidates from different ethnic groups applied. Whereas white candidates applied to degree programmes with initial rejection rates of 47.2 per cent on average, the corresponding figures for candidates from all ethnic minority groups are around 10 percentage points higher. This suggests that a tendency, already noted, on the part of ethnic minority applicants to apply to more numerically competitive programmes is likely to form at least part of the explanation for their lower offer rates compared to their white counterparts.

Column 4 of Table 1 reports the average percentage of ethnic minority applicants to particular degree subject areas at particular institutions. Here we see that whereas white candidates apply to degree subject areas at institutions where ethnic minorities make up less than one-fifth of all applicants on average, ethnic minority candidates apply to degree programmes where ethnic minorities make up around one-third of all applicants on average. This confirms that some degree subject areas at some Russell Group universities attract significantly greater numbers of ethnic minority applicants than would be expected simply by chance. The correlation between the percentage of ethnic minority applicants to particular subject areas at particular institutions and the numerical competitiveness variable is only moderately strong, at *r* = 0.445, suggesting that both variables may help to explain ethnic differences in the chances of receiving an offer from a Russell Group university.

Table 2 reports the results of a multivariate analysis of the data using binary logistic regression models to compare the odds of an application to a Russell Group university being met with an offer of admission rather than being rejected if the candidate is from an ethnic minority background rather than from the white group. Model 1 includes only ethnicity and year of application as predictor variables and so the results essentially replicate what was seen previously in Table 1: namely that the odds of receiving an offer from a Russell Group university rather than being rejected are less than half as favourable for applicants classified as black Caribbean, black African, Pakistani, Bangladeshi and Other than for applicants classified as white (0.35 to 1, 0.23 to 1, 0.36 to 1, 0.38 to 1 and 0.47 to 1, respectively). The odds are also less than perfectly equitable for Indian (0.63 to 1), Chinese (0.82 to 1) and Mixed ethnicity (0.76 to 1) applicants relative to their white counterparts.

**Table 2.** Comparative odds of an offer of admission from a Russell Group university.

	Model 1 Controls for year of application	Model 2 Plus controls for other applicant characteristics	Model 3 Plus controls for prior attainment	Model 4 Plus controls for numerical competitiveness	Model 5 Plus interaction with percentage of ethnic minority applicants
**Ethnic group (White British)**					
Black Caribbean	0.35***	0.51***	0.61***	0.76***	0.70***
Black African	0.23***	0.38***	0.45***	0.54***	0.55***
Pakistani	0.36***	0.41***	0.51***	0.64***	0.66***
Bangladeshi	0.38***	0.44***	0.51***	0.74***	0.79***
Indian	0.63***	0.61***	0.62***	0.84***	0.81***
Chinese	0.82***	0.85***	0.72***	0.86***	0.89**
Mixed	0.76***	0.80***	0.80***	0.88***	0.87***
Other	0.47***	0.54***	0.59***	0.46***	0.73***
**School type (Private)**					
State grammar		1.19***	1.14***	0.98	0.99
State non-selective		0.67***	0.86***	0.80***	0.80***
**Local HE participation rate (Top quintile)**					
4th quintile		0.95***	0.98	0.95***	0.95***
3rd quintile		0.85***	0.92***	0.91***	0.91***
2nd quintile		0.85***	0.95***	0.88***	0.88***
Bottom quintile		0.75***	0.89***	0.77***	0.77***
**Female**		0.90***	1.00	1.25***	1.26***
**Mature applicant**		0.28***	0.73***	1.03	1.03
**Application timing (UCAS main deadline)**					
Early (by 15th October)		0.63***	0.60***	0.90***	0.90***
Late (after 15th January)		0.41***	0.39***	0.44***	0.44***
**A-level grades**					
No. of A* grades			1.46***	1.61***	1.61***
No. of A grades			1.29***	1.40***	1.39***
No. of B grades			1.20***	1.18***	1.18***
No. of C grades			0.95***	0.89***	0.89***
No. of D grades			0.74***	0.71***	0.71***
No. of E grades			0.68***	0.65***	0.64***
**Tariff point equivalent to A-levels**					
420+ (A*A*A* or higher)			1.37***	2.58***	2.70***
360–419 (AAA to A*A*A)			0.52***	0.87**	0.90
300–359 (BBB to AAB)			0.41***	0.71***	0.74***
240–299 (CCC to BBC)			0.25***	0.40***	0.41***
<240 (CCD or lower)			0.36***	0.51***	0.52***
**Facilitating subjects at A-level**					
Biology			0.89***	1.20***	1.19***
Chemistry			0.96***	1.09***	1.09***
English Literature			0.88***	0.89***	0.90***
Geography			1.32***	1.13***	1.13***
History			1.07***	1.01	1.02
Languages			1.21***	1.07***	1.08***
Mathematics			1.11***	1.06***	1.05***
Physics			1.48***	1.14***	1.14***
**Numerical competitiveness**				0.948***	0.947***
**% ethnic minority applicants × White British**					1.006***
× Black Caribbean					1.002
× Black African					0.991***
× Pakistani					0.988***
× Bangladeshi					0.988***
× Indian					0.996*
× Chinese					0.990***
× Mixed					0.995**
× Other					0.996*
Pseudo *R*^2^	0.023	0.075	0.130	0.270	0.271
Chi-square	4741	15801	27234	56610	56730

*Note*: Figures reported are odds ratios. Asterisks indicate statistical significance at the *p* < 0.10 (*), *p* < 0.05 (**) and *p* < 0.01 (***) levels. All models control for year of application.

Model 2 of Table 2 separates out ethnic differences in admissions offer chances from those attributable to other applicant characteristics including school type, the higher education participation rate in their local area, their sex, whether or not they are a mature student, and the timing of their application in the annual admissions cycle. Here it can be seen that the comparative odds of receiving an offer from a Russell Group university are lower for applicants from non-selective state schools relative to private schools (0.67 to 1), for those from areas with lower local HE participation rates, for females relative to males (0.90 to 1), for mature applicants as compared to those from the traditional 18–21 age group (0.28 to 1), and for those who apply early or late compared to those who meet UCAS’s main application deadline (0.63 to 1 and 0.41 to 1, respectively). Notably, controlling for these other social background characteristics helps explain some of the disparity in admissions chances between white applicants and their black Caribbean, black African, Pakistani, Bangladeshi and Other ethnicity counterparts, as can be seen from the fact that the odds ratios for these groups move closer towards 1.

Model 3 of Table 2 adds controls for applicants’ prior attainment. Here we see that the odds of receiving an offer from a Russell Group university are improved by having a greater number of A*, A and B grades at A-level (1.46 to 1, 1.29 to 1 and 1.20 to 1, respectively) or by having a very high tariff point equivalency score for applicants who have entry qualifications other than A-level (1.37 to 1). Offer chances are also higher for A-level applicants who had studied Geography, History, Languages, Mathematics or Physics. These controls for applicants’ prior attainment help to further explain ethnic group differences in the odds of an offer from a Russell Group university. However, after controlling for prior attainment, the odds of receiving an offer from a Russell Group university remain considerably lower for black Caribbean (0.61 to 1), black African (0.45 to 1), Pakistani (0.51 to 1) and Bangladeshi (0.51 to 1) applicants, and for applicants from Indian (0.62 to 1), Chinese (0.72 to 1), Mixed (0.80 to 1) and Other ethnic backgrounds (0.59 to 1), relative to comparably qualified white applicants.

Model 4 of Table 2 adds a control for the numerical competitiveness of the applicants’ chosen degree subject areas at their chosen institutions as measured by initial rejection rates. As expected, applying to a course that is more numerically competitive reduces the odds of receiving an offer of a place (0.948 to 1). Importantly, controlling for numerical competitiveness appreciably reduces the extent of ethnic group differences in the odds of receiving an offer from a Russell Group university, indicating that ethnic minority applicants have lower offer rates than comparably qualified white applicants partly because they are more likely to apply to oversubscribed courses. However, even after controlling for numerical competitiveness, substantially lower comparative odds of receiving an offer persist for all ethnic minority applicants, especially those from black Caribbean (0.76 to 1), black African (0.54 to 1), Pakistani (0.64 to 1) and Bangladeshi (0.74 to 1) backgrounds.

Finally, Model 5 of Table 2 tests the hypothesis that ethnic inequalities in admissions chances widen as the percentage of ethnic minority applicants increases. In this model, applicants’ ethnicity is interacted with the percentage of ethnic minority applicants to their chosen degree subject areas at their chosen institutions. Whereas an increase in the percentage of ethnic minority applicants improves the odds of an offer from a Russell Group university for white applicants (1.006 to 1), an increase in the percentage of ethnic minority applicants widens the disparity in admissions chances between white applicants and those from black African (0.991 to 1), Pakistani (0.988 to 1), Bangladeshi (0.988 to 1), Chinese (0.990 to 1) and Mixed (0.995 to 1) ethnic backgrounds. Negative interaction effects are also evident for applicants from the Indian (0.996 to 1) and Other (0.996 to 1) ethnic groups, although these are statistically significant only at the *p* < 0.10 level. For black Caribbean applicants, in contrast, no statistically significant interaction effect is observed, possibly because of the relatively small sample size for this group.

**Figure 1.**
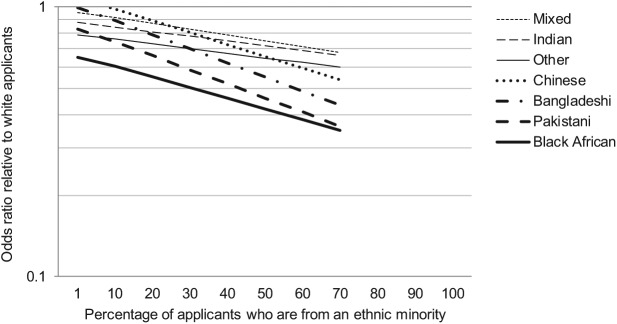
Association between the percentage of ethnic minority applicants and the degree of ethnic inequality in the odds of receiving an offer from a Russell Group University. *Note*: The slopes are truncated at 70 on the x-axis to reflect the actual range of values in the data.

These interaction effects are displayed pictorially in Figure 1, where it can clearly be seen that ethnic disparities in admissions chances are at their smallest when the percentage of ethnic minority applicants is so small that it approaches zero. However, as the percentage of ethnic minority applicants increases, the comparative odds of an offer for ethnic minority applicants decrease sharply relative to white applicants. These patterns are consistent with the hypothesis that when ethnic minorities apply to courses at rates that exceed their representation in the population at large, admissions selectors at Russell Group are rejecting ethnic minority applicants at disproportionately high rates, perhaps in order to achieve a more ethnically representative student body.

## Discussion and Conclusions

This article has reported two major findings. First, that while it is true that ethnic minority applicants are more likely than white applicants to choose oversubscribed courses, ethnic minority applicants remain less likely to receive offers from Russell Group universities than comparably qualified white applicants even after the numerical competitiveness of courses has been taken into account. But might this be a spurious finding? As was noted earlier, due to data limitations, applicants’ prior attainment has been measured using actual rather than predicted A-level grades data; controls have been included for eight generally ‘facilitating’ subjects rather than the specific combination of A-level subjects required for the courses to which applicants had applied; information on applicants’ grades at GCSE could not be included at all; and the numerical competitiveness of courses has been measured with reference to relatively broad subject areas rather than specific degree programmes. If Russell Group applicants from ethnic minority backgrounds are more likely than white applicants with the same actual A-level grades to have their A-level grades under-predicted; if they are less likely to have the combination of A-level subjects required for entry to their chosen degree programme; if they tend to have poorer GCSE grades; and if they tend to apply to the most oversubscribed courses within the broader subject categories distinguished here; then the extent to which ethnic minority applicants are disadvantaged in the competition for places at Russell Group universities relative to comparably qualified white applicants will have been overstated in the results presented above.

However, in-house analysis carried out by UCAS, which has not been published but was summarized briefly in the *Times Higher Education* (Grove, 2013), found that even after controlling for predicted A-level grades, GCSE performance, specific A-level subjects studied, and specific degree programme applied to, a ‘small’ but still significant ethnic bias in admissions chances persists. Given this, it would seem fairly certain that ethnic inequalities in the chances of admission to Russell Group universities are not entirely or even largely explained by ethnic differences in propensities to apply to the most oversubscribed courses. To be certain of this, however, further quantitative analysis of more detailed data needs to be carried out so that clearly relevant variables, such as the numerical competitiveness of an applicant’s chosen course, can be measured more precisely, and so that potentially important omitted variables, such as an applicant’s grades at GCSE, can be taken into account. This would require restrictions on the supply of UCAS data to third-party researchers to be lifted, so that appropriately anonymized but sufficiently detailed data could be made available for independent analysis. It is deeply concerning, then, that UCAS decided recently that it would no longer supply any individual-level data whatsoever to third-party researchers (UCAS, 2014), a decision that has since been challenged by the Social Mobility Commission (Machin, 2015).

The second major finding of this article is that ethnic inequalities in admissions chances are greater for degree subject areas at Russell Group universities where the percentage of ethnic minority applicants is higher. Once again, the question arises: could this be a spurious result? One possibility is that the finding merely reflects how the average ‘quality’ of ethnic minority applicants to particular courses declines as the percentage of ethnic minority applicants increases. However, this assumes that more applicants necessarily means weaker applicants, and that admissions selectors can reliably distinguish between stronger and weaker applicants from among those with the same A-level grades.^3^ Again, determining whether or not this is the case would require further analysis of more detailed data on the full range of selection criteria used. Until then at least, we need to take seriously the finding presented here, that ethnic minority applicants are less likely than comparably qualified white applicants to receive offers from Russell Group universities, especially in relation to degree programmes that attract disproportionately high numbers of ethnic minority applicants.

Although the reason for this second major finding cannot be established with the data at hand, a plausible explanation for the observed pattern is that, consciously or unconsciously (Equality Challenge Unit, 2013), some admissions selectors are unfairly rejecting some ethnic minority applicants in order to achieve an entering class with an ethnic mix that is ultimately representative of, say, the wider national population. Because ethnic minorities apply in disproportionately high numbers for certain courses at certain institutions, the goal of ultimate representativeness is inevitably at odds with a concern for equal treatment during the admissions process. This is highly problematic since the 2010 Equality Act expressly forbids the unequal treatment of individual applicants on the basis of ethnicity, including as a result of the use of quotas to determine the number of places available to applicants from different ethnic origins (Equality Challenge Unit, 2012; Equality and Human Rights Commission, 2012).

More research is clearly needed to establish to what extent admissions selectors are conscious of the ethnic origins of applicants and whether this affects their decision-making. Experimental methods could be used to explore whether ethnic disparities in offer rates are diminished when UCAS forms are anonymized to conceal the ethnic backgrounds of applicants (as gender disparities in orchestra hiring rates were diminished in an experiment involving ‘blind’ auditions; Goldin and Rouse, 2000). Research of a more qualitative nature is also needed to explore what principles of evaluation guide admissions decision-making in UK universities and how the potentially conflicting goals of ultimate representativeness and equal treatment are rationalized and reconciled. This work could profitably involve a qualitative analysis of universities’ published policies on admissions in the vein of recent work on the ‘discourses of fair access’ (Archer, 2007; Bowl and Hughes, 2013; McCaig and Adnett, 2009; Stevenson et al., 2010), as well as in-depth interviews with a wide cross-section of admissions selectors to build on the insights offered by smaller scale pioneer studies (Burke and McManus, 2011; Zimdars, 2010).

Lastly, but no less importantly, the universities themselves could do more to help explain the stark ethnic disparities in offer rates that remain after A-level grades, facilitating subjects and the numerical competitiveness of courses are taken into account. There is considerable scope for universities, either individually or collectively, to conduct detailed analyses of their own admissions data; to undertake thorough reviews of their own admissions policies and practices; to publish their findings openly and transparently; and to commit to making the changes required for a fairer and more equitable admissions system.

## Notes

1. This use of the category ‘black’ conflates several different ethnic identities and does not distinguish between British citizens and citizens of other nations. In the empirical component of this article, I make use of a more detailed categorization of ethnicity and focus specifically on UK-based applicants. However, it is important to recognize that even the most detailed ‘official’ ethnicity categories conceal linguistic, religious and other differences and may not be very meaningful to the people they are meant to describe (see Aspinall, 2002).

2. Ethnic disparities in offer rates have been shown to exist at other Old (pre-1992) and New (post-1992) universities too, although they are smaller in magnitude than is the case for Russell Group universities (see Boliver, 2015, for further details).

3. It is worth noting that the same line of argument would lead us to expect to see school-type disparities in the chances of receiving an offer from a Russell Group university diminish as the percentage of private school applicants increases and their average ‘quality’ falls in a similar fashion. An analysis of the UCAS dataset (results not presented but available from the author on request) shows that this hypothesis is not borne out empirically: private school applicants are more likely to receive offers from Russell Group universities than comparably qualified applicants from non-selective state schools and the size of the disparity is the same irrespective of the percentage of applicants who were from private schools.
